# Dehydroepiandrosterone enhances decidualization in women of advanced reproductive age

**DOI:** 10.1016/j.fertnstert.2017.12.024

**Published:** 2018-04

**Authors:** Douglas A. Gibson, Ioannis Simitsidellis, Olympia Kelepouri, Hilary O.D. Critchley, Philippa T.K. Saunders

**Affiliations:** a MRC Centre for Inflammation Research, University of Edinburgh, Edinburgh, United Kingdom; b MRC Centre for Reproductive Health, University of Edinburgh, Edinburgh, United Kingdom

**Keywords:** DHEA, decidualization, aging, fertility, androgens

## Abstract

**Objective:**

To investigate the impact of the androgen precursor dehydroepiandrosterone (DHEA) on the decidualization of human endometrial stromal cells isolated from women of advanced reproductive age.

**Design:**

In vitro study.

**Setting:**

University research institute.

**Patient(s):**

Proliferative phase primary human endometrial stromal fibroblasts (hESFs) were isolated from women of advanced reproductive age (n = 16; mean age, 44.7 ± 2.3). None of the women were receiving hormone therapy or had endometriosis.

**Intervention(s):**

Isolated hESFs were decidualized in vitro by incubation with P (1 μM) and cAMP (0.1 mg/mL) in the presence, or absence, of DHEA (10 nM, 100 nM).

**Main Outcome Measure(s):**

Secretion of androgens was assessed by ELISA. Expression of decidualization markers and endometrial receptivity markers was assessed by quantitative polymerase chain reaction and ELISA.

**Result(s):**

Decidualization responses were retained in hESF isolated from women of advanced reproductive age. Supplementation with DHEA increased androgen biosynthesis and concentrations of T and dihydrotestosterone were ∼3× greater after coincubation with DHEA compared with hESF stimulated with decidualization alone. Addition of DHEA to decidualized hESF increased expression of the decidualization markers IGFBP1 and PRL and the endometrial receptivity marker SPP1. DHEA enhanced secretion of IGFBP1, PRL, and SPP1 proteins maximally by day 8 of the decidualization time course concomitant with peak androgen concentrations.

**Conclusion(s):**

These novel results demonstrate DHEA can enhance in vitro decidualization responses of hESF from women of advanced reproductive age. Supplementation with DHEA during the receptive phase may augment endometrial function and improve pregnancy rates in natural or assisted reproductive cycles.

**Discuss:** You can discuss this article with its authors and other readers at **https://www.fertstertdialog.com/users/16110-fertility-and-sterility/posts/28813-24930**

Establishment of pregnancy requires hormone-dependent priming of the endometrium to promote an environment that is receptive to an implanting blastocyst. Pregnancy rates decline with increasing maternal age. Women above the age of 40 represent the most rapidly growing age group trying to conceive (1), however, lower rates of pregnancy are reported in women aged over 40 compared with those under 40 (2). Reductions in ovarian reserve explain some of this age-related decline in fertility as live-birth rates (LBRs) using donor oocytes from young fertile donors are comparable among women up until the age of 44 (3), while decline in LBR in autologous IVF cycles is apparent from age 36 onward (4). Implantation rate is a sensitive and accurate measure of endometrial function in assisted reproductive technology (ART) cycles. Notably, a decline in implantation rates has been reported in oocyte donor ET cycles in recipients over the age of 50 (5), a trend that is detectable from age 40 onward and has been reported in some cohorts to be significant from the mid-40s 3, 6 and as early as age 40 (7). Thus, despite advances in ART, age remains a critical determinant of fertility and implantation is a rate-limiting step in achieving a successful pregnancy.

Endometrial receptivity and implantation are dependent on endometrial stromal cell decidualization, which plays a critical role in defining a time-limited window of receptivity when embryo implantation is favored. Decidualization is accompanied by biosynthesis of androgens (8) that in turn support the biosynthesis of factors critical to the development of a robust decidualization response and associated endometrial receptivity (8). Appropriate regulation and integration of these intracrine/paracrine signals is key to promoting an endometrial microenvironment that can support establishment and maintenance of pregnancy.

Dehydroepiandrosterone (DHEA) is an adrenal androgen precursor that is abundant in the circulation. In women, the androgen receptor agonists T and dihydrotestosterone (DHT) are synthesized within peripheral tissues from DHEA by the actions of the enzymes 3β- and 17β-hydroxysteroid dehydrogenases (3BHSD, 17BHSD) and 5α-reductases. All these enzymes are expressed in the normal endometrium (9). Whether changes in the bioavailability of DHEA determine the capacity for androgen biosynthesis in the premenopausal endometrium is not known. Importantly, the circulating “precursor pool” of DHEA declines with age, and concentrations of DHEA in women ages 40–50 are half those of women in their 20s (10). In addition, circulating concentrations of DHEA are elevated in women with polycystic ovarian syndrome (PCOS) (11), but the potential impact of increased DHEA on endometrial function is poorly understood. We therefore speculated that changes in circulating DHEA might alter endometrial androgen bioavailability and modify the endometrial microenvironment.

In the current study we assessed the impact of DHEA supplementation on decidualization of endometrial stromal fibroblasts isolated from women of advanced reproductive age. Our results demonstrate that DHEA can enhance decidualization and endometrial receptivity by acting as precursor to androgens produced within the endometrium. Thus, targeted supplementation with DHEA may enhance endometrial function and improve pregnancy rates in women of advanced reproductive age.

## Materials and methods

### Human Studies

**Supplemental Figure 1:**
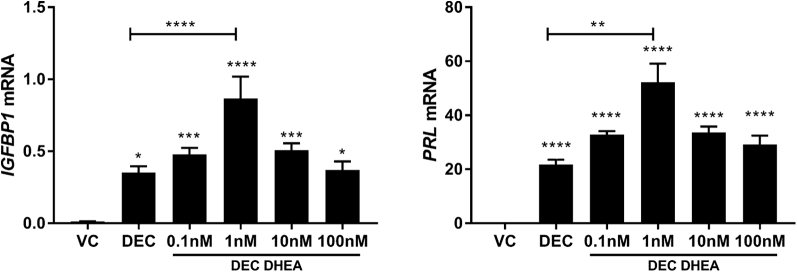
Dose-dependent impact of dehydroepiandrosterone (DHEA) on expression of decidualization markers. To determine the impact of varying concentrations of DHEA on decidualization, immortalized human endometrial stromal cells (SHT290) were treated with either vehicle control (VC), decidualization (DEC) media, or DEC media supplemented with 0.1, 1, 10, or 100nM DHEA (DEC DHEA) for 4 days. Expression of mRNAs encoding *IGFBP1* and *PRL* were determined by quantitative polymerase chain reaction. n = 3, triplicate treatments. One-way ANOVA with Tukey's multiple comparisons test (IGFBP1) or Kruskal-Wallis with Dunn's multiple comparison test (PRL). *P<.05; ***P<.001; ****P<.0001.

Primary human endometrial tissue (proliferative phase, n = 16) was obtained from women of advanced reproductive age (mean age, 44.7 ± 2.3) undergoing surgery for nonmalignant gynecological conditions. None of the women were receiving hormone therapy or had endometriosis. Primary human endometrial stromal fibroblasts (hESFs) were isolated from proliferative phase endometrium, and the cycle phase was determined as reported previously (12). Decidualization was induced by addition of decidualization (DEC) media (RPMI 1640, 2% charcoal-stripped fetal calf serum [FCS], 0.1 mg/mL 8-Br-cAMP [Sigma, B5386], 1 μM P [Tocris, cat no. 2835]). Some cell cultures were supplemented with DHEA (10 or 100 nM, Santa Cruz sc-202573) for the duration of the culture period. Control cultures were incubated with RPMI 1640, 2% charcoal-stripped FCS, and equivalent volume of vehicle control (DMSO). To assess the time-dependent accumulation of secreted products, treatments were maintained for the duration of each time point. hESFs were treated for 1, 2, 4, and 8 days as indicated. The expression of decidualization markers and an androgen-regulated receptivity marker (SPP1) was assessed by quantitative polymerase chain reaction (qPCR) and ELISA. Secretion of T, DHT, SPP1, IGFBP1, and PRL was assessed by ELISA. Some experiments were performed in immortalized human endometrial stromal cells (SHT290) as indicated in Supplemental Figure 1. Culture conditions, treatments, and sample collection/analysis for SHT290 cells were identical to hESF cultures.

### Measurement of mRNA

Isolation of mRNAs, preparation of cDNAs, and analysis by qPCR was performed according to standard protocols (12); samples were quantified by standard curve method or by the comparative ΔΔCt method with CYC as an internal control. Primers/probes are given in Supplemental Table 1.

### ELISA

T, DHT, insulin-like growth factor binding protein 1 (IGFBP1), and PRL were measured in culture supernatants as described previously (8) (Supplemental Tables 2–4). Secretion of SPP1 was quantified by bead ELISA (R&D) according to the manufacturer's instructions and measured using a Bio-Plex 200 HTF machine and Bio-Plex Manager Software (ver. 5, BioRad).

### Western Blot Analysis

Western blotting was performed using 50 μg/lane total cell lysates; membranes were probed with rabbit anti-AKR1C3 (Supplemental Table 5; predicted molecular weight 37 kDa; green), loading control was goat anti-actin (Supplemental Table 5; predicted molecular weight 43 kDa; red). Membranes were incubated with species-specific fluorescent-conjugated secondary antibodies and visualized using the Licor Odyssey system (Licor). Protein bands detected by Western blot were quantified by performing densitometry analysis using ImageJ (NIH.gov). Data were normalized to actin and expressed as fold change relative to control treatment.

### Statistics

Statistical analysis was performed using Graphpad prism. Two-way analysis of variance (ANOVA) was used to determine significance between treatments in grouped data. Nonparametric testing was used where sample sizes were insufficient to confirm normality of data distribution; the Kruskal-Wallis test was used to assess differences between treatments. Where data were analyzed as fold change, significance was tested using one-sample *t*-test and a theoretical mean of 1. *P*<.05 was considered statistically significant. All data are presented as mean ± SEM.

### Study Approval

Written informed consent was obtained from all subjects before surgery; ethics approval was granted by the Lothian Research Ethics Committee (LREC 10/S1402/59). Methods were carried out in accordance with NHS Lothian Tissue Governance guidelines.

## Results

### DHEA Supplementation Increases Intracrine Androgen Production

**Figure 1:**
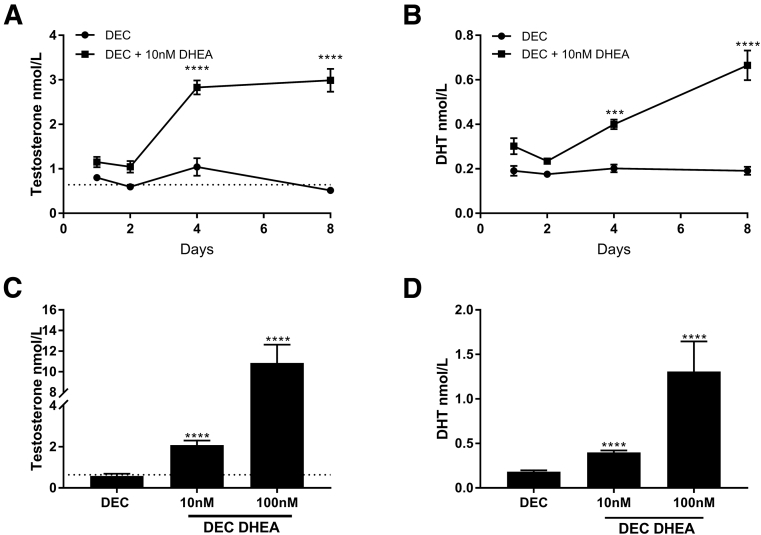
Dehydroepiandrosterone (DHEA) increases androgen synthesis during decidualization. Human endometrial stromal fibroblasts (HESFs) were treated with either vehicle control (VC), decidualization (DEC) media, or DEC media supplemented with 10 nM DHEA (DEC DHEA) (**A, B**) or DEC media supplemented with either 10 or 100 nM DHEA (**C, D**). (**A, B**) Secretion of both T and dihydrotestosterone (DHT) was detected in DEC groups at all time points and significantly increased in DEC DHEA (supplemented with 10 nM DHEA) compared with DEC alone. Neither T nor DHT was detected in hESFs treated with VC (not shown). (**C, D**) HESFs were decidualized for 4 days and supplemented with 10 nM or 100 nM DHEA. T and DHT were significantly increased in a dose-dependent manner with increasing DHEA concentrations. Dotted lines show circulating concentrations of T in women (Table 1). n = 6–16 patients, duplicate treatments. Two-way ANOVA (**A, B**) or Kruskal-Wallis test (**C, D**). ***P<.001; ****P<.0001.

**Figure 2:**
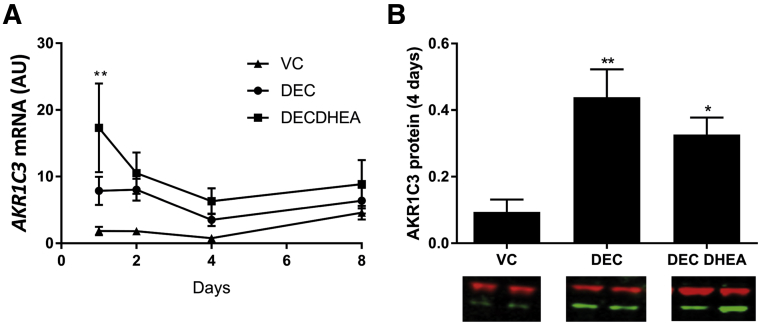
Increased androgen production is driven by precursor availability and not changes in enzyme expression. (**A**) Concentrations of mRNAs encoding *AKR1C3* were assessed by quantitative polymerase chain reaction at 1, 2, 4, and 8 days of treatment. Expression of AKR1C3 increased over time with decidualization (DEC) treatment but was not changed by coincubation with dehydroepiandrosterone (DHEA), although at day 1 DEC DHEA was increased compared with vehicle control (VC) (n = 6; *P*<.01). (**B**) Concentrations of AKR1C3 protein were assessed by Western blot in Human endometrial stromal fibroblast lysates after 4 days and were significantly increased with DEC (*P*<.01) and DEC DHEA (*P*<.05) treatment compared with controls but were not different between DEC and DEC DHEA. n = 4–5 patients, duplicate treatments. *P<.05; ** *P*<.01. AU = arbitrary units.

We assessed secretion of androgens by hESF from day 1 to 8 of a decidualization time course in the presence or absence of 10 nM DHEA (Fig. 1A and 1B). This concentration was chosen to mimic those reported for premenopausal women below the age of 40 (Table 1). At each time point assessed, supplementation of DEC media with DHEA significantly increased the amount of androgen secreted into the culture media such that by days 4 and 8 concentrations of T in DHEA supplemented cultures were ∼3× those of hESF incubated in standard DEC medium (Fig. 1A; *P*<.0001). DHT concentrations were also increased by supplementation with DHEA, being 2× higher after 4 (*P*<.001) and further increased after 8 days (*P*<.0001) compared with DEC alone (Fig. 1B). We next assessed whether secretion of androgens in hESF was influenced by the amount of DHEA by supplementing with 10 or 100 nM DHEA (Fig. 1C and 1D). The concentration of both T and DHT secreted by hESF was robustly and significantly increased in a dose-dependent manner by both 10 nM (*P*<.0001) and 100 nM (*P*<.0001) DHEA compared with DEC alone (Fig. 1C and 1D). DHEA did not affect expression of aldo-keto-reductase 1C3 (AKR1C3), the key enzyme required for the conversion of precursors to T during decidualization of hESF (8) (Fig. 2), suggesting the increase in T biosynthesis in DHEA supplemented cultures was due to an increase in the amount of precursor steroid and not altered concentrations of enzyme.

**Table 1:** Average serum androgen concentrations reported in pre- and postmenopausal women and in women with PCOS.

Variable	Age range	DHEA	A4	T	DHT	n	Reference
Premenopausal	23–32	7.1	5.9	0.3		49	(11)
	23–49			0.6	0.3	31	(13)
	27–38	13.0	4.3	0.9	0.5	10	(14)
	<40		2.5	0.7		48	(15)
Average		10.1	4.2	0.6	0.4	138	
PCOS	24–36	14.1	26.8	0.7		114	(11)
	18–38	17.4	7.1	1.7		52	(16)
	30.0 ± 4.4	22.5	5.5	1.5	0.4	74	(17)
Average		18.0	13.1	1.3	0.4	240	
Postmenopausal	42–72			0.4	0.1	19	(13)
	54–65	5.7	2.3	0.9	0.4	5	(14)
	40–59			1.6	0.6	41	(15)
Average		5.7	2.3	1.0	0.4	65	

*Note:* All hormone concentrations are stated in nmol/L. A4 = androstenedione; DHEA = dehydroepiandrosterone; DHT = dihydrotestosterone; PCOS = polycystic ovary syndrome.

### DHEA Supplementation Enhances Decidualization and Expression of the Endometrial Receptivity Marker SPP1

**Figure 3:**
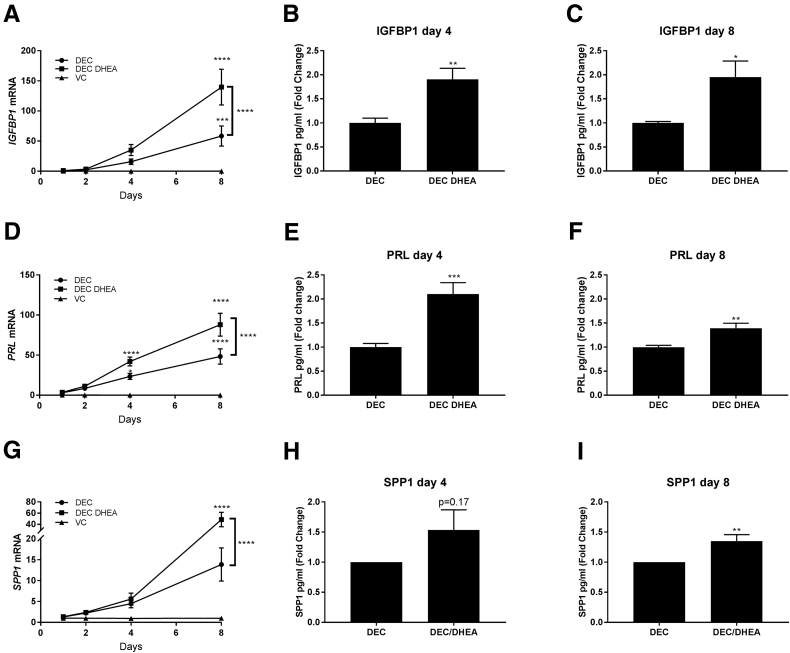
Dehydroepiandrosterone (DHEA) supplementation enhances expression of decidualization and endometrial receptivity markers. Human endometrial stromal fibroblasts were treated as in Figure 1A and 1B, and expression of IGFBP1, PRL, and SPP1 was assessed by quantitative polymerase chain reaction and ELISA. Concentrations of mRNAs encoding *IGFBP1* (**A**), *PRL* (**D**), and *SPP1* (**G**) were assessed by quantitative polymerase chain reaction; expression of all three markers increased over time with decidualization (DEC) treatment and were enhanced by DHEA treatment, which was apparent after 4 days and most pronounced by 8 days of treatment. Cotreatment with DHEA significantly increased secretion of IGFBP1 (**B, C**) and PRL (**E, F**) compared with DEC alone. Secretion of SPP1 protein (**H** and **I**) was significantly increased with DEC DHEA after 8 days of treatment. n = 6 patients, duplicate treatments. Two-way ANOVA (**A, D, G**), or one-sample *t* test (**B, C, E, F, H, I**). *P<.05; **P<.01; ***P<.001; ****P<.0001.

To assess whether decidualization was enhanced by addition of DHEA, the expression of the decidualization markers IGFBP1 and PRL was determined by ELISA and qPCR. Time course analysis revealed a significant time-dependent increase in expression of *IGFBP1* (Fig. 3A; *P*<.01) and *PRL* (Fig. 3D; *P*<.0001) in hESFs treated with DEC. Cotreatment with DHEA further enhanced expression of mRNAs encoding both *IGFBP1* (*P*<.001) and *PRL* (*P*<.0001) by 8 days compared with DEC alone (Fig. 3A and 3D). Assessment of supernatants revealed a significant increase in the secretion of IGFBP1 and PRL protein with DEC consistent with previous studies (Supplemental Fig. 1) (8). Addition of DHEA led to a robust and significant increase in secretion of decidualization markers, which was approximately double that of DEC alone for IGFBP1 (Fig. 3B; *P*<.01) and PRL (Fig. 3E; *P*<.001) after 4 days, an effect that was still detectable after 8 days treatment for both IGFBP1 (Fig. 3C; *P*<.001) and PRL (Fig. 3F; *P*<.01). Since androgen production was dependent on DHEA concentration during decidualization, we also performed a DHEA dose-response experiment to determine the impact of DHEA concentration on decidualization (Supplemental Fig. 1). We tested DHEA concentrations, which ranged from subphysiological (0.1 nM), to physiological (1, 10 nM), and supraphysiological (100 nM DHEA). We found that the impact of DHEA on expression of decidualization markers was dose dependent, appearing to elicit a bell-shaped dose response consistent with androgens acting as “Goldilocks factors” (18). Notably, neither very low nor very high concentrations of DHEA reduced expression of the decidualization markers IGFBP1 and PRL. The greatest increase in expression of decidualization markers was detected after supplementation with DHEA concentrations between 1 and 10 nM in line with reported physiological serum concentrations in healthy women (Table 1).

Secreted phosphoprotein 1 (SPP1; osteopontin) is an essential marker of endometrial receptivity, and its expression increases as hESFs decidualize (8). Our previous study (8) identified that SPP1 was androgen regulated during decidualization. In the current study, time course analysis revealed that expression of SPP1 was increased by 8 days after stimulation with DEC media (Fig. 3G). Addition of DHEA had a time-dependent impact on SPP1 mRNA with a striking and significant increase on day 8 compared with DEC alone (*P*<.001; Fig. 3G). Concentrations of secreted SPP1 protein were measured by ELISA in matched supernatants from 4 and 8 days of treatment. SPP1 concentrations were variable among patients (Supplemental Fig. 1). Fold-change analysis of SPP1 concentrations between DEC and DEC DHEA revealed no significant change after 4 days of treatment (Fig. 3H) but a significant increase in cultures treated with DHEA after 8 days (Fig. 3I; *P*<.01).

## Discussion

Optimizing endometrial responses is critical for pregnancy success. To date, there has been limited evidence to suggest that altering the bioavailability of androgen precursors like DHEA could have an impact on endometrial function. An older study reported that the endometrium can convert DHEA to active androgens and that formation of T is increased during the secretory phase (19), but whether this affects hESF function is not known. Pharmacological concentrations of DHEA (100 μM) impair decidualization of hESF by inhibiting glucose flux and the pentose phosphate pathway (20); however, 100 nM DHEA is reported to enhance expression of the decidualization marker Homeobox A10 (HOXA10) and reduce production of reactive oxygen species in murine endometrial stromal fibroblasts (21). In the current study, we provide the first evidence that DHEA can act as a prohormone to increase intracrine androgen production in hESFs derived from normal endometrium during decidualization. Furthermore, we demonstrate that the capacity for a robust decidualization response is retained in women of advanced reproductive age and can be enhanced by increasing availability of the androgen precursor DHEA. SPP1, the only factor identified that is common to all reported endometrial receptivity gene sets (22), was increased maximally after supplementation with DHEA after 8 days. Concomitantly, we detected an increase in expression of the decidualization markers IGFBP1 and PRL. Increased expression of these key markers was associated with maximal production of androgens in the DHEA-treated group. Taken together, these results suggest that poor endometrial responses in some older women may reflect deficits in the bioavailability of systemic factors, such as DHEA, rather than functional decline of the cells/tissue per se. These findings support and expand evidence that androgens have the capacity to fine-tune the trajectory of endometrial tissue remodeling and thus targeting androgen action may be beneficial in improving suboptimal endometrial responses.

Fluctuations in DHEA that occur as a result of endocrinopathies provide some insights into the potential impact of DHEA on fertility. PCOS is a complex, multifactorial disorder that is characterized by the presence of two out of three criteria: anovulation, polycystic ovarian morphology, and hyperandrogenism (Rotterdam criteria) (23). DHEA, as well as androstenedione and T, is reported to be elevated in PCOS (11), however, interpreting the impact(s) of hyperandrogenism on endometrial function is challenging. Elevated expression of androgen receptor has been reported in the endometrium of women with PCOS (24), and decidualization is reported to be impaired in hESF isolated from women with PCOS in some but not all patients (25). In the current study, the highest concentration of DHEA tested (100 nM), which exceeded reported serum concentrations for PCOS (18 nM; Table 1), enhanced production of T and DHT during decidualization but did not alter expression of decidualization markers. Thus, although androgen signaling is altered in women with PCOS, its impact on endometrial function is as yet unclear. A recent study that compared IVF outcomes in 364 women with tubal factor infertility to 307 women with PCOS reported that women with PCOS had more oocytes retrieved and higher clinical pregnancy and LBRs (26). It is conceivable that higher concentrations of androgens may have an impact on endometrial function in women with PCOS, which may account for some of the differences in IVF success, however, further studies are needed to determine any potential endometrial effect. Future studies should focus on investigating the responses of hESFs isolated from women undergoing donor egg recipient regimen (E_2_ and P_4_ before ET). This approach would account for menstrual cycle disturbances as a result of ovarian dysfunction and allow isolated assessment of the impact of age and DHEA on endometrial responses.

Implantation and pregnancy rates are reported to remain constant with increasing recipient age when donor oocytes are used in ART cycles. Reports from collated Centers for Disease Control data using large patient cohorts from multiple centers in the Unites States (n = 17,339 cycles) demonstrated no age-dependent decline in pregnancy rates of recipients under the age of 50 (5). The most up-to-date data from the Society for Assisted Reproductive technology member clinic survey from 27,959 fresh donor oocyte IVF cycles reported a small but significant drop in pregnancy rates in recipient women over the age of 44; but it was noted that absolute LBRs were still relatively high in this patient cohort (3). In the absence of oocyte donation, age-related decline in IVF success rates is reported (4). A number of different strategies have been reported to help improve success of non–oocyte donor IVF cycles including supplementation with DHEA, however, studies investigating the impact of DHEA on reproductive outcomes have been limited by a lack of randomized controlled trials. A recent Cochrane review examining the effect of either direct androgen supplementation (T) or DHEA supplementation in women undergoing ART reported that androgens may be associated with improved LBRs but had no conclusive effect on ovarian function or miscarriage rates (27). Barad et al. investigated the impact of DHEA on pregnancy rates in women with diminished ovarian reserve and reported improved clinical pregnancy rates compared with controls (28). However, in this study half of the pregnancies reported occurred spontaneously, before IVF, which we believe could be due to improved endometrial function/receptivity. An increased capacity to convert supplemented DHEA to T was also associated with improved pregnancy rates in a study of 213 women with diminished ovarian reserve undergoing IVF, consistent with a role for adrenal precursors in successful pregnancies (29). Although these studies suggest DHEA supplementation may improve pregnancy rates, the mechanism and impact of DHEA on endometrial function were not explored.

Sex steroid precursors represent new therapeutic targets in both natural and assisted reproduction. While there is accumulating evidence that DHEA supplementation is well tolerated in postmenopausal women, the effects in premenopausal women are poorly defined. Notably, the Cochrane review on androgen supplementation in ART concluded there was still insufficient data to draw conclusions on the safety of either T or DHEA supplementation (27). We propose that future trials should focus on evaluating the efficacy of transient targeted delivery rather than systemic longitudinal treatment to reduce the likelihood of side effects. Indeed, it has already been reported that intravaginal DHEA does not have systemic effects (30).

### Conclusion

The novel findings presented in the current study provide compelling evidence that DHEA can act as a precursor to intracrine androgen production in the premenopausal endometrium and that concentrations of DHEA within the physiological range enhance decidualization. Our data suggest that decidualization responses are retained in women of advanced reproductive age but that age-dependent reductions in circulating concentrations of DHEA may constrain endometrial function. We suggest that supplementation with DHEA may therefore represent a novel therapeutic strategy that could enhance fertility in women of advanced reproductive age.

## Figures and Tables

**Supplemental Figure 2:**
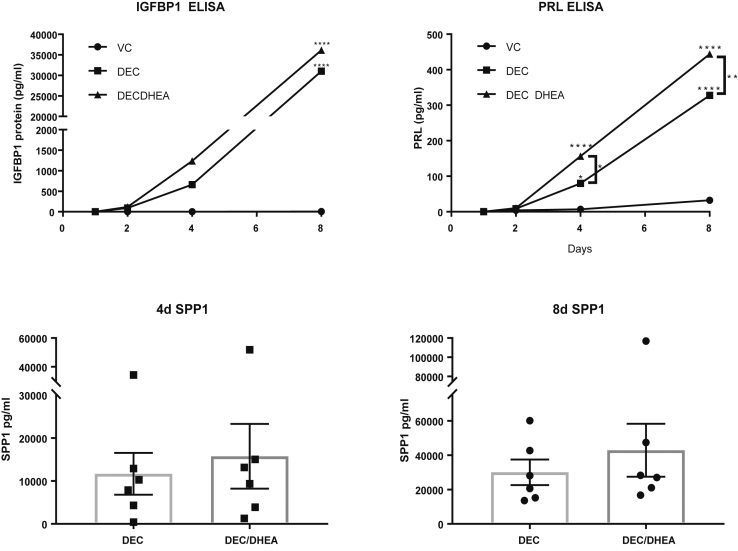
DHEA supplementation enhances secretion of decidualization and endometrial receptivity markers. Primary hESF were treated with either vehicle control (VC), DEC media, or DHEA-supplemented DEC media (DEC DHEA) for up to 8 days. Concentrations of IGFBP1, PRL, and SPP1 were measured by ELISA. Median IGFBP1 and PRL concentrations plotted at each time point and treatment. SPP1 was measured by ELISA in day 4 and day 8 supernatants from hESF treated with DEC and DEC DHEA. n = 6 patients, duplicate treatments. Two-way ANOVA. *P<.05; **P<.01; ***P<.001; ****P<.0001.
