# Supplementary material for: Dehydroepiandrosterone enhances decidualization in women of advanced reproductive age
Source: Fertil Steril. 2018 Apr;109(4):728–734.e2. doi: 10.1016/j.fertnstert.2017.12.024 (PMC5908781; doi:10.1016/j.fertnstert.2017.12.024)
Supplement: Supplemental Tables 1–5 [file mmc1.docx]

*Supplemental Table 1* - Primers and probes for qRTPCR.

| **Gene Symbol** | **5' to 3'** | **3' to 5'** |
| --- | --- | --- |
| *AKR1C3* | tgggttccgccatatagatt | tcgatgaaaagtggaccaaa |
| *IGFBP1* | aatggattttatcacagcagacag | aatggattttatcacagcagacag |
| *PRL* | aaaggatcgccatggaaag | gcacaggagcaggtttgac |
| *SPP1* | gagggcttggttgtcagc | caattctcatggtagtgagttttcc |

*Supplemental Table 2* – Assay performance

| ELISA | Intra-assay CV (%) | Inter-assay CV (%) | Sensitivity |
| --- | --- | --- | --- |
| IGFBP1 | 2.4-10.2 | 5.5-8.7 | 31.2 pg/ml |
| Testosterone | 6.6-9.6 | 6.1-8.5 | 0.022 ng/ml |
| Dihydrotestosterone | 3.9-11.4 | 5.9-12.1 | 6.0 pg/ml |

*Supplemental Table 3* - Cross-reactivity of testosterone ELISA

| **Steroid** | **% Cross-Reactivity** |
| --- | --- |
| Testosterone | 100 |
| 5α-DHT | 5.2 |
| Androstenedione | 1.4 |
| Androstanediol | 0.8 |
| Progesterone | 0.5 |
| Androsterone | 0.1 |

*Supplemental Table 4* – Cross-reactivity of dihydrotestosterone ELISA

| **Steroid** | **% Cross-Reactivity** |
| --- | --- |
| Dihydrotestosterone | 100 |
| Testosterone | 8.7 |
| 5β-dihydrotestosterone | 2.0 |
| Androstenedione | 0.2 |

*Supplemental Table 5 – antibodies used in Western blot analysis*

| Antibody name | Species raised | Supplier | Dilution |
| --- | --- | --- | --- |
| AKR1C3 | Rabbit | Abcam (Ab137546) | 1:500 |
| Actin | Goat | Santa-Cruz (sc-1616) | 1:500 |
